# Soluble klotho as a marker of renal fibrosis and podocyte injuries in human kidneys

**DOI:** 10.1371/journal.pone.0194617

**Published:** 2018-03-28

**Authors:** Nam-Jun Cho, Dong-Jae Han, Ji-Hye Lee, Si-Hyong Jang, Jeong Suk Kang, Hyo-Wook Gil, Samel Park, Eun Young Lee

**Affiliations:** 1 Department of Internal Medicine, Soonchunhyang University Cheonan Hospital, Cheonan, Korea; 2 Department of Pathology, Soonchunhyang University Cheonan Hospital, Cheonan, Korea; Center for Molecular Biotechnology, ITALY

## Abstract

Klotho deficiency is relevant to renal fibrosis and podocyte injury *in vivo* and *in vitro*. We examined whether histological findings of renal biopsy specimens were associated with the levels of soluble klotho in humans. We investigated renal biopsy specimens of 67 patients and detailed microscopic findings were reviewed. Soluble serum/urinary klotho and urinary angiotensinogen were assessed by enzyme-linked immunosorbent assays, and tissue klotho expression was assessed by immunohistochemical staining. The median age of the study participants was 35.6 years. High serum klotho levels (≥14 pg/mL) were associated with decreased odds ratios (ORs) of interstitial fibrosis (OR = 0.019, *P* = 0.003) and segmental sclerosis (OR = 0.190, *P* = 0.022) in multivariable logistic regression analysis. Patients with a lower urinary klotho-to-creatinine ratio (UKCR) were significantly more likely to have diffuse foot process effacement (OR = 0.450, *P* = 0.010). The area under the receiver-operating characteristic curve (AUC) of serum klotho for predicting interstitial fibrosis was 0.920 (95% CI, 0.844–0.996), and the best cut-off value of serum klotho was 138.1 pg/mL. The AUC of UKCR for predicting diffuse foot process effacement was 0.754 (95% CI, 0.636–0.872), and the best cut-off value of UKCR was 96.7 pg/mgCr. Urinary angiotensinogen-to-creatinine ratio was not associated with serum klotho, UKCR, or any pathological finding. Our data suggested that soluble serum and urinary klotho levels represent a potential biomarker to predict renal fibrosis and podocyte injury in humans.

## Introduction

The *klotho* gene was first identified as an anti-aging gene in 1997 [1]. Klotho is a type I single-pass transmembrane protein expressed in various organs, and is especially abundant in the kidney [2]. Membranous klotho has an important role in phosphate metabolism by acting as a co-receptor with fibroblast growth factor (FGF) receptors for the FGF23 ligand [3]. Klotho is released as a soluble and secreted form by alternative splicing of the *klotho* gene or by cleavage of large extracellular domain of transmembrane klotho [4, 5]. Soluble klotho acts as a hormonal factor to inhibit cellular apoptosis, anti-oxidation, inhibition of fibrosis, and modulation of ion transport [6–9].

Soluble klotho is present in blood, urine and cerebrospinal fluid, and is quantified by enzyme-linked immunosorbent assays [10, 11]. Reduced serum and urinary klotho levels are observed in the early stages of acute kidney injury and chronic kidney disease (CKD), with progressive decline in more advanced stages [12–14]. Progression of renal diseases is commonly associated with renal fibrosis, which is histologically evident as interstitial fibrosis/tubular atrophy (IFTA) and glomerulosclerosis [15, 16]. Several animal models reported that klotho deficiency triggers renal fibrosis via inhibition of growth factors, enhanced endoplasmic reticulum stress, up-regulation of transient receptor potential channels, and up-regulation of the renin-angiotensin system (RAS) [17–20]. However, no human studies have examined the association between soluble klotho and renal fibrosis, and the exact role of soluble klotho in renal fibrosis in humans is unknown.

Reduced blood klotho concentration is also associated with increased albuminuria, especially in patients with diabetes [21, 22]. Although klotho is mainly expressed in renal tubules, and more abundantly in the distal convoluted tubules, a recent study reported that the glomerulus is another source of klotho expression [23]. In the same study, administration of recombinant klotho protected against slit diaphragm disruption induced by the overexpression of TRPC6 in mice. In addition to renal fibrosis, it is unclear whether podocyte foot process changes are correlated with soluble klotho level.

In the present study, we tested the hypothesis that histological findings of renal biopsy specimens are associated with serum/urine soluble klotho levels. Urinary angiotensinogen levels were also evaluated to explore the role of RAS with soluble klotho in renal fibrosis. We report that serum and urinary klotho levels are strongly correlated with renal fibrosis and foot process effacement of podocyte, respectively. The results support the translation of *in vivo* and *in vitro* data to clinical settings.

## Materials and methods

### Study population and study design

Between July 2009 and August 2011, 70 patients who underwent renal biopsy at the Soonchunhyang University Cheonan Hospital (Cheonan, Republic of Korea) were enrolled. Three patients including two who had inadequate biopsy specimens and one who was diagnosed with renal cell carcinoma, were excluded. A renal biopsy specimen was considered adequate if it contained ≥4 glomeruli. The study was conducted in accordance with the 1975 Declaration of Helsinki and with the 2000 revision, and was approved by the Institutional Review Board (IRB) of the Soonchunhyang University Cheonan Hospital. The need for informed consent was waived by the IRB, as the current study was considered a retrospective review of anonymized clinical data.

### Clinical and laboratory information

Baseline clinical characteristics determined the day before renal biopsy included age, gender, weight, height, body mass index (BMI), diabetes mellitus (DM), hypertension (HTN), urinary protein-to-creatinine ratio (UPCR), blood urea nitrogen, serum creatinine, estimated glomerular filtration rate (eGFR), serum calcium, serum phosphorus, serum albumin, total cholesterol, and triglyceride (TG) level. The eGFR was calculated using the Chronic Kidney Disease Epidemiology Collaboration equation [24].

### Pathology

All renal biopsy specimens (*n* = 67) were reviewed and confirmed by two independent pathologists (JH Lee, SH Jang). Biopsy specimens were prepared according to standard protocols for light microscopy, immunofluorescence, and electron microscopy (for details, see S1 Appendix). The percentage of glomeruli affected by segmental or global glomerulosclerosis was assessed by dividing the number of sclerosing glomeruli with the total number of acquired glomeruli. The degree of foot process effacement was assessed based on the percentage of evaluated areas and grouped as follows: absent, 0–10%; focal mild, 10–30%; focal moderate, 30–50%; focal severe, 50–70%; and diffuse, ≥70%. IFTA was evaluated semi-quantitatively according to the proportion of the cortical area involved, and grouped as follows: absent, <1%; mild, 1–25%; moderate, 25–50%; and severe, ≥50% of the total area. Intimal thickening was assessed as the percentage of narrowing lumen of the most severely affected blood vessel, and classified as follows: absent, 0%; mild, <25%; moderate, 25–50%; and severe, ≥50%.

The expression of klotho in renal biopsy specimens was evaluated using immunohistochemistry (IHC). Tissue sections (2-μm thickness) were cut, deparaffinized in xylene, and hydrated using an ethanol-deionized water series. Endogenous peroxidase activity was blocked using 3% H_2_O_2_ in methanol for 15 min. Sections were washed and stained with a 1:200 dilution of rabbit polyclonal antibody against klotho (LS-B6625; LifeSpan BioSciences, Seattle, WA, USA). Primary antibody binding was detected using Bond Polymer Refine Detection Kit (Leica, Wetzlar, Germany). Slides were counterstained with hematoxylin.

Renal klotho expression was semi-quantitatively calculated using H-score method which is commonly used for IHC evaluation [25]. Briefly, predominant cytoplasmic staining intensities were designated as negative (0), weak (1+), or strong (2+) and the percentage of distal tubule cells at each staining intensity level was calculated. Finally, an H-score was assigned using the following formula: 2 × percentage of strongly staining cells + 1 × percentage of weakly staining cells, giving a possible range of 0–200. All cases with discrepancy in IHC scoring were reanalyzed and discussed between both pathologists.

### ELISA of soluble klotho and urinary angiotensinogen

Blood samples were centrifuged for 15 min at 3,000 rpm within 30 min of collection. The serum was extracted and stored at -70°C until analysis. Urine samples were centrifuged for 10 min at 3,000 rpm to remove particulate matter and stored at -70°C until analysis. Serum and urinary concentrations of klotho were analyzed by Human Soluble alpha-klotho Assay Kits (Immuno-Biological Laboratories, Gunma, Japan) and urinary angiotensinogen concentrations were measured with a Human Total Angiotensinogen Assay Kit (Immuno-Biological Laboratories, Gunma, Japan) according to the manufacturer’s instructions. All samples were analyzed twice and the intra- and inter-assay coefficients of variation were <10%. The levels of urinary klotho or angiotensinogen were expressed as the ratio of urinary klotho or angiotensinogen-to-urinary creatinine to assess the patients’ hydration and renal function.

### Statistical analysis

Statistical analyses were performed using R version 3.4.0 (The R Foundation for Statistical Computing, Vienna, Austria). Serum klotho, urinary klotho, and urinary angiotensinogen values were missing in 13, 2, and 9 patients respectively. Thus, each analysis was performed after excluding participants with missing values. Categorical variables were expressed as counts (percentage), normally distributed continuous variables as means ± SD, and non-normally distributed continuous variables as medians (interquartile ranges). Significantly skewed variables were logarithmically transformed where possible. Differences between groups were analyzed by Mann-Whitney U test or Kruskal-Wallis tests according to the number of groups. Pearson’s correlation coefficient or Spearman’s rank correlation coefficient was used to test the correlation between individual continuous variables. In multiple comparisons, pairwise Mann-Whitney U test with Bonferroni correction was performed between the groups. Categorical variables were analyzed using the Pearson's Chi-squared test or Fisher’s exact test as appropriate. Multivariable logistic regression was performed to identify the association between soluble klotho and pathologic features after adjustment for possible confounders. To measure the sensitivity and specificity of serum/urinary klotho at different cut-off values, a conventional ROC curve was generated. Best cut-off values were determined by selecting the data point that maximized the sum of sensitivity and specificity. *P*-value < 0.05 was regarded as statistically significant and two-tailed tests were performed in all hypothesis tests.

## Results

### Baseline characteristics

The median age of 67 study participants was 35.6 (19.9–48.5) years. Nineteen (27.5%) patients had HTN and 3 (4.3%) patients had DM. The median values of eGFR and UPCR were 109.4 (79.8–130.3) mL/min/1.73 m^2^ and 545.4 (range, 193.5–2032.0) mg/gCr. The median serum klotho level was 14.0 (7.0–122.2) pg/mL, the median urinary klotho-to-creatinine ratio (UKCR) was 76.6 (range, 12.1–197.2) pg/mgCr, and the median urinary angiotensinogen-to-creatinine ratio (UAngCR) was 17.1 (8.4–27.3) ng/mgCr.

### Renal biopsy findings

In the renal biopsy specimens of these patients, immunoglobulin A (IgA) nephropathy was diagnosed in 26 (38.8%) patients, focal segmental glomerulosclerosis in 12 (17.9%) patients, minimal change disease in 7 (10.4%) patients, membranous nephropathy and thin basement membrane disease in 3 (4.5%) patients, membranous proliferative glomerulonephritis in 2 (3.0%) patients, and post-infectious glomerulonephritis, lupus nephritis, crescentic glomerulonephritis, acute tubular necrosis, amyloidosis were diagnosed in 1 (1.5%) patient each. The specimens of 9 (13.4%) patients showed nonspecific findings. There was no significant correlation between soluble klotho level and pathological diagnosis.

Detailed microscopic findings are presented in Table 1.

**Table 1:** Microscopic findings of the renal biopsies.

Microscopic findings	Grade	Number (%)
**Glomerular changes**		
Globally sclerosed glomeruli	Absent	30 (44.8)
	<25%	24 (35.8)
	25–50%	7 (10.5)
	≥50%	6 (9.0)
Segmentally sclerosed glomeruli	Absent	29 (43.3)
	<25%	19 (28.4)
	25–50%	11 (16.4)
	≥50%	8 (11.9)
Podocyte foot process effacement	Absent	6 (9.0)
	Focal mild	14 (20.9)
	Focal moderate	8 (11.9)
	Focal marked	13 (19.4)
	Diffuse	26 (38.8)
**Tubulointerstitial changes**		
Tubular atrophy	Absent	18 (26.9)
	Mild	40 (59.7)
	Moderate	6 (9.0)
	Severe	3 (4.5)
Interstitial fibrosis	Absent	18 (26.9)
	Mild	40 (59.7)
	Moderate	7 (10.5)
	Severe	2 (3.0)
**Vascular changes**		
Intimal thickening	Absent	30 (44.8)
	Mild	33 (49.3)
	Moderate	2 (3.0)
	Severe	2 (3.0)

Mean number of glomeruli (n = 67) was 21.0 ± 10.3. The median percentage of globally sclerosed glomeruli was 4.9 (0–18.4) % and the median percentage of segmentally sclerosed glomeruli was 5.3 (0–33.3) %.

### Association of pathological findings with serum klotho levels

**Fig 1:**
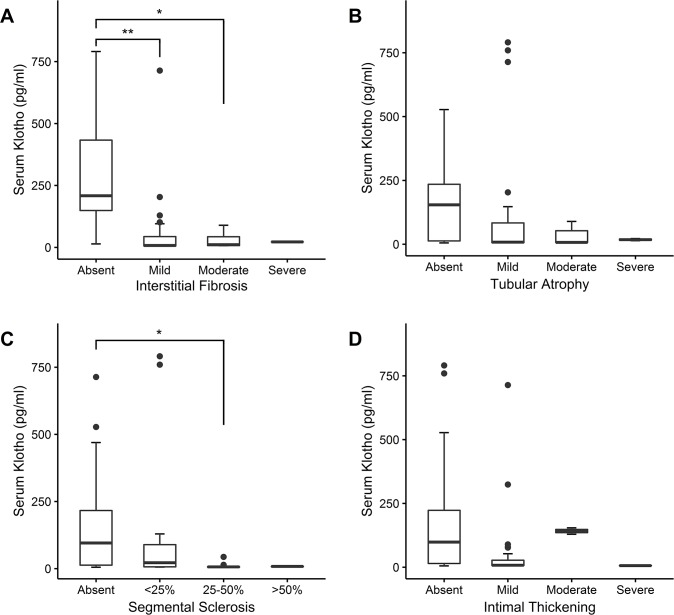
Comparison of serum klotho levels in different groups for each pathology. Boxplots for serum klotho levels are presented according to the severity of each pathologic finding: (A) Interstitial fibrosis, (B) tubular atrophy, (C) segmental sclerosis of glomeruli, and (D) intimal thickening of arterial wall. ^*^P < 0.01; ^**^P < 0.001.

Serum klotho levels varied with the degree of segmental glomerulosclerosis (*P* = 0.003), tubular atrophy (*P* < 0.001), interstitial fibrosis (*P* < 0.001), and arterial intimal thickening (*P* = 0.02). Serum klotho levels in the absence of interstitial fibrosis (208.5 [148.9–433.2] pg/mL) were significantly higher than in mild (7.5 [6.1–43.5] pg/mL) and moderate interstitial fibrosis (10.9 [7.3–43.0] pg/mL). Serum klotho levels in the absence of segmental glomerulosclerosis (95.5 [12.9–216.4] pg/mL) were significantly higher than in moderate segmental glomerulosclerosis (6.4 [5.3–7.1] pg/mL) (Fig 1).

**Fig 2:**
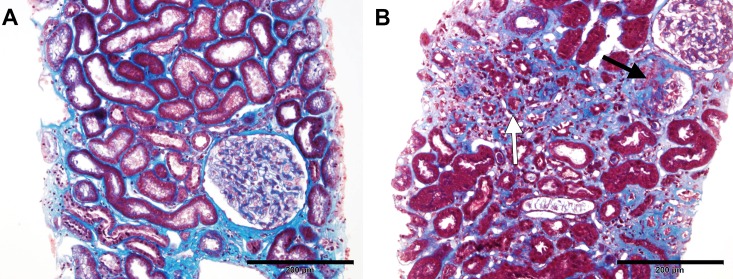
Representative images of Masson’s trichrome-stained kidney sections according to serum klotho levels. (A) The specimen from 68-year-old male patient with IgA nephropathy and serum klotho level of 790.9 pg/mL showed nearly intact interstitium and glomerulus. (B) The specimen from a 38-year-old male patient with IgA nephropathy and serum klotho level of 6.4 pg/mL showed marked interstitial fibrosis and tubular atrophy (white arrow), and sclerosing glomerulus (black arrow). Scale bar = 200 μm.

Patients were divided into binary groups according to their serum klotho levels. The cut-off point was selected based on the median value (14 pg/mL). Baseline demographics and clinical characteristics of the two groups are presented in Table 2. No significant differences in parameters were detected between the two groups. Multivariable logistic regression was performed adjusting for other variables that potentially affect outcomes. If serum klotho level was high (≥14 pg/mL), the odds ratios (ORs) of interstitial fibrosis (OR = 0.019, *P* = 0.003) and segmental sclerosis (OR = 0.190, *P* = 0.022) were decreased after adjustment for age, gender, BMI, DM, HTN, eGFR, and log UPCR (Table 3). Representative light microscopic images of patients in the high and low serum klotho groups are presented in Fig 2.

**Table 2:** Baseline demographics and clinical characteristics according to serum klotho level.

	Low serum klotho	High serum klotho	
	(< 14 pg/ml, *n* = 27)	(≥ 14 pg/ml, *n* = 27)	*P*-value
Klotho range, pg/ml	5.2–13.9	14.0–790.9	–
Age, years	37.3 (28.9–54.8)	34.7 (19.3–45.1)	0.223
Sex, male (%)	14 (51.9)	16 (59.3)	0.584^a^
Hypertension, present (%)	7 (25.9)	6 (22.2)	0.750^a^
Diabetes, present (%)	1 (3.7)	2 (7.4)	1.000^b^
BMI	23.4 ± 3.3	24.2 ± 4.5	0.450
Albumin, g/dl	3.93 ± 0.90	3.75 ± 1.04	0.652
Urea nitrogen, mg/dl	14.5 (11.6–20.6)	12.4 (10.8–19.0)	0.489
Creatinine, mg/dl	0.9 (0.7–1.1)	0.8 (0.7–0.9)	0.524
Calcium, mg/dl	8.69 ± 0.70	8.54 ± 0.72	0.457
Phosphorus, mg/dl	3.86 ± 0.63	3.93 ± 0.91	0.756
Total cholesterol, mg/dl	207.0 (171.5–290.0)	220.0 (158.5–279.5)	0.678
Triglycerides, mg/dl	120.0 (99.0–176.5)	144.0 (86.0–208.5)	0.622
eGFR, ml/min per 1.73 m^2^	96.2 (77.6–124.5)	115.7 (81.0–130.3)	0.272
UPCR, mg/gCr	425.0 (216.4–1912.1)	780.0 (213.4–2867.5)	0.539

Data are presented as mean ± SD, median (interquartile range), or count (%) as appropriate. *P*-values are calculated by

^a^Pearson’s Chi-squared test or

^b^Fisher’s exact test for categorical variables, and by Mann–Whitney U test for continuous variables. BMI, body mass index; eGFR, estimated glomerular filtration rate; UPCR, urine protein to creatinine ratio.

**Table 3:** Association of serum klotho level with interstitial fibrosis and segmental sclerosis.

		Presence of interstitial fibrosis		Presence of segmental sclerosis	
	Low serum klotho	High serum klotho	*P*-value	High serum klotho	*P*-value
Model 1^a^	1 (reference)	0.041 (0.002–0.240)	0.003	0.228 (0.072–0.738)	0.016
Model 2^b^	1 (reference)	0.035 (0.002–0.244)	0.004	0.228 (0.059–0.783)	0.023
Model 3^c^	1 (reference)	0.019 (0.001–0.173)	0.003	0.190 (0.041–0.746)	0.022

Presented as odds ratio (95% confidence interval) attained by multivariable logistic regression. Serum klotho levels were categorized into low and high serum klotho group according to their median values (14 pg/mL).

^a^Model 1: not adjusted

^b^Model 2: adjusted for age, gender, and body mass index

^c^Model 3: adjusted for Model 2 variables plus diabetes mellitus, hypertension, estimated glomerular filtration rate, and log urine protein-to-creatinine ratio

### Association of pathological findings with urinary klotho levels

**Fig 3:**
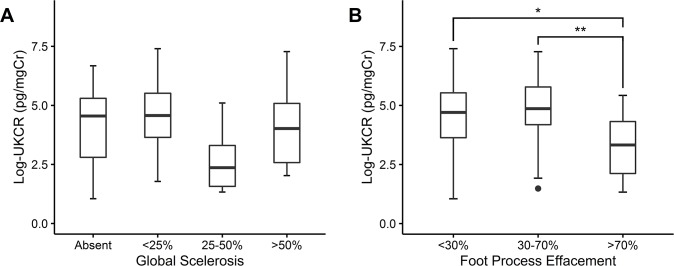
Association of log-transformed urinary klotho-to-creatinine ratio with global sclerosis and foot process effacement. Boxplots for log-transformed urinary klotho-to-creatinine ratio are shown according to the severity of each pathological finding: (A) Global glomerular sclerosis, (B) foot process effacement of podocyte. ^*^P < 0.05; ^**^P < 0.01. UKCR: urinary klotho-to-creatinine ratio.

Baseline demographics and clinical characteristics of 65 patients and relationship with urinary klotho are presented in Table 4. Log-transformed UKCR was used because of normal distribution after the transformation. Baseline eGFR levels were significantly associated with urinary klotho levels (*R* = 0.273, *P* = 0.028) and DM participants had lower log UKCR levels (1.8 ± 0.4) compared with non-DM participants (4.3 ± 1.6, *P* = 0.02) (Table 4). The log UKCR levels differed according to the degree of global glomerulosclerosis (*P* = 0.033) and foot process effacement of podocyte (*P* = 0.009). The log UKCR levels among the groups of global glomerulosclerosis did not show a consistent trend; patients who had ≥70% of foot process effacement (3.3 ± 1.3 pg/mgCr) revealed less log UKCR levels than patients who had <30% of foot process effacement (4.5 ± 1.8 pg/mgCr) and 30–70% of foot process effacement (4.8 ± 1.6 pg/mgCr) (Fig 3).

**Table 4:** Baseline demographics and clinical characteristics based on urinary klotho-to-creatinine ratio.

	Values (N = 65)	rho	*P*-value
Log UKCR, pg/mgCr	4.15 ± 1.67	–	–
Age, years	35.6 (19.9–48.5)	-0.051	0.685
Sex, male	39 (58.2)	–	0.056^a^
Hypertension, present	18 (26.9)	–	0.799^a^
Diabetes, present	3 (4.5)	–	0.020^a^
BMI	23.6 ± 3.9	0.107	0.394
Albumin, g/dl	3.88 ± 0.99	0.134	0.286
Urea nitrogen, mg/dl	14.5 (11.2–19.6)	-0.155	0.217
Creatinine, mg/dl	0.8 (0.7–1.0)	-0.150	0.233
Calcium, mg/dl	8.64 ± 0.68	0.090	0.475
Phosphorus, mg/dl	3.86 ± 0.82	0.093	0.460
Total cholesterol, mg/dl	203.0 (155.5–273.5)	-0.127	0.312
Triglycerides, mg/dl	141.0 (98.5–192.5)	0.002	0.986
eGFR, ml/min per 1.73 m^2^	109.4 (79.8–130.3)	0.273	0.028
UPCR, mg/gCr	545.4 (193.5–2032.0)	-0.232	0.063

Data are presented as mean ± SD, median (interquartile range), or count (%) as appropriate. Pearson correlation coefficient or Spearman’s rank correlation coefficients with *P*-values are presented for the continuous variables.

^a^*P*-values of Mann–Whitney U test are presented for the categorical variables. UKCR: urinary klotho-to-creatinine ratio; BMI: body mass index; eGFR: estimated glomerular filtration rate; UPCR: urinary protein-to-creatinine ratio.

**Fig 4:**
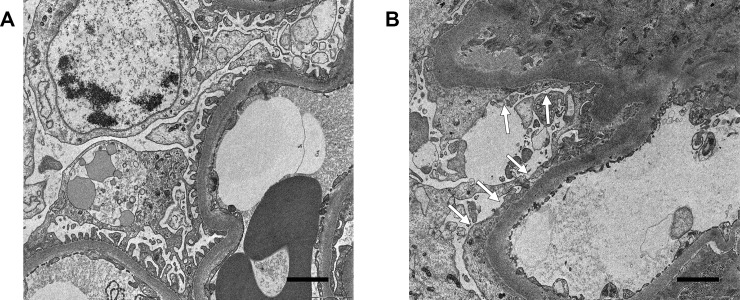
Representative transmission electron microscopy images according to urinary klotho-to-creatinine ratio. (A) The specimen obtained from a 55-year-old male patient with IgA neprhopathy and a urinary klotho-to-creatinine ratio of 30.83 pg/mgCr showed preserved foot process of podocyte. (B) The specimen obtained from a 58-year-old male patient with IgA nephropathy and a urinary klotho-to-creatinine ratio of 0.44 pg/mgCr showed diffuse effacement of foot process (arrows). Scale bar = 5 μm.

Multivariable logistic regression was performed to assess the relationship between urinary klotho levels and the degree of foot process effacement after adjusting for other variables. After adjustment for age, gender, BMI, DM, HTN, eGFR, and interstitial fibrosis, the OR of diffuse foot process effacement was decreased as each pg/mgCr of log UKCR was increased (OR = 0.450, *P* = 0.010) (Table 5). Representative transmission electron microscopy images of patients according to the level of urinary klotho-to-creatinine ratio are presented in Fig 4.

**Table 5:** Association of each pg/mgCr increase in log urinary klotho-to-creatinine ratio and diffuse foot process effacement.

	Diffuse effacement of foot process	
Log UKCR	Odds ratio	*P*-value
Model 1^a^	0.558 (0.374–0.790)	0.002
Model 2^b^	0.460 (0.256–0.733)	0.003
Model 3^c^	0.450 (0.224–0.778)	0.010

Presented as odds ratios (95% confidence interval) based on multivariable logistic regression. Foot process effacement of podocyte was grouped according to absent/focal effacement (effacement <70%) and diffuse effacement (effacement ≥70%). Log-transformed urinary klotho-to-creatinine ratio was treated as a continuous variable (pg/mgCr). UKCR: urinary klotho-to-creatinine ratio.

^a^Model 1: not adjusted

^b^Model 2: adjusted for age, gender, and body mass index

^c^Model 3: adjusted for Model 2 variables plus diabetes mellitus, hypertension, estimated glomerular filtration rate, and interstitial fibrosis.

### Serum/urinary klotho as a biomarker predicting renal pathology

**Fig 5:**
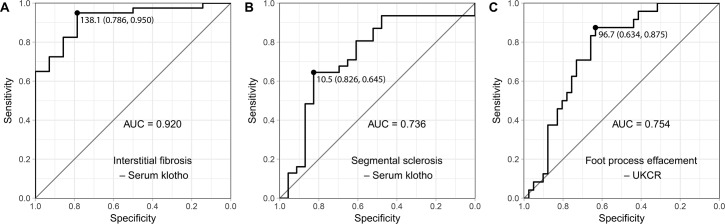
Receiver-operating characteristic (ROC) analyses for predicting renal pathology. ROC curves are shown according to the predictors and outcomes: (A) ROC curve for predicting interstitial fibrosis based on serum klotho, (B) ROC curve for predicting segmental sclerosis based on serum klotho, and (C) ROC curve for predicting foot process effacement based on the urinary klotho-to-creatinine ratio. Best cut-off values were presented as black circles and certain values (with specificity and sensitivity). AUC, area under the ROC curve; UKCR, urinary klotho-to-creatinine ratio.

The AUC of serum klotho was 0.920 (95% CI, 0.844–0.996) for predicting interstitial fibrosis (Fig 5A), and 0.736 (95% CI, 0.592–0.880) for predicting segmental sclerosis (Fig 5B). The best cut-off value of serum klotho was 138.1 pg/mL (sensitivity, 0.950; specificity, 0.786), for predicting interstitial fibrosis. It was 10.5 pg/mL (sensitivity, 0.645; specificity, 0.826), for predicting segmental sclerosis. A cut-off value 138.1 pg/mL also yielded good sensitivity (sensitivity, 0.935; specificity, 0.478) for predicting segmental sclerosis.

The AUC of UKCR for predicting diffuse foot process effacement was 0.754 (95% CI, 0.636–0.872) (Fig 5C). The best cut-off value of UKCR for predicting diffuse foot process effacement was 96.7 pg/mgCr (sensitivity, 0.875; specificity, 0.634).

### Association of urinary angiotensinogen levels with serum/urinary klotho levels

There was no clear association of UAngCR with serum klotho (rho = -0.088, *P* = 0.563) and UKCR (rho = 0.535, *P* = 0.689). Further, no significant differences were detected in UAngCR levels with the pathological findings. Compared with baseline demographics and clinical characteristics, UAngCR was significantly correlated with the patient’s age (rho = 0.574, *P* < 0.001), serum albumin (rho = -0.405, *P* = 0.002), serum calcium (rho = -0.477, *P* < 0.001), serum total cholesterol (rho = 0.408, *P* = 0.002), serum TG (rho = 0.313, *P* = 0.017), eGFR (rho = -0.355, *P* = 0.006), and UPCR (rho = 0.321, *P* = 0.014). The UAngCR levels varied with the degree of HTN (*P* = 0.036).

### IHC staining for klotho protein

**Fig 6:**
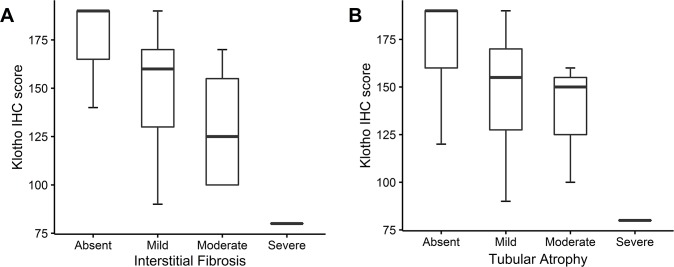
Comparison of the klotho immunohistochemistry (IHC) staining score based on each pathologic finding. Boxplots for klotho IHC staining score are shown according to the severity of each pathologic finding: (A) Interstitial fibrosis and (B) tubular atrophy. IHC staining score for distal tubule were calculated as: percentage of strongly stained distal tubule × 2 + percentage of weakly stained distal tubule.

**Fig 7:**
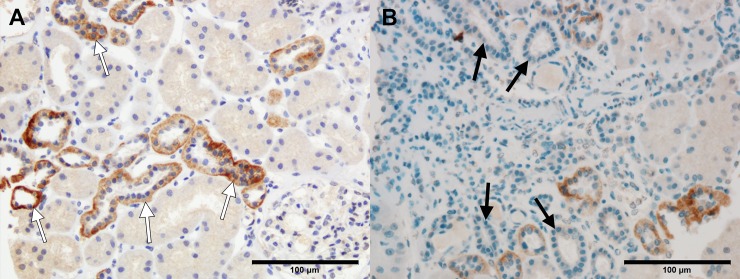
Representative images of immunohistochemistry (IHC) staining for klotho based on adjacent pathological features. (A) Distal tubules surrounded by normal tubuloinsterstitium showed strong IHC staining for klotho (white arrows). (B) Distal tubules surrounded by inflamed interstitium and atrophic tubules showed absent or weak IHC staining for klotho (black arrows). Scale bar = 100 μm.

We assessed klotho expression in the kidneys of 29 patients using an anti-klotho antibody. Klotho was expressed abundantly in the distal tubules of all patients. Expression was evident in the proximal tubules of 3 patients. The median IHC staining score for distal tubule was 160.0 (120.0–170.0). IHC staining score for distal tubule was decreased as either interstitial fibrosis or tubular atrophy progressed, but there was no statistical significance (Fig 6). A detailed review of each slide revealed distal tubules surrounded by inflamed or fibrotic interstitium displaying absent or weaker IHC staining compared with tubules surrounded by intact interstitium (Fig 7). The IHC staining score for distal tubule was not correlated with serum klotho level (rho = -0.044, *P* = 0.128) and urinary klotho-to-creatinine ratio (rho = -0.096, *P* = 0.627).

## Discussion

The progression of renal diseases is characterized by renal fibrosis, which histologically presents as IFTA and glomerulosclerosis [15, 16]. The fibrotic process within the tubulointerstitium and glomeruli involves infiltration of inflammatory cells, activation of fibroblasts and myofibroblasts, and epithelial-mesenchymal transition [26]. Klotho, an anti-aging protein, is an emerging target of renal fibrosis progression. Klotho exhibits anti-fibrotic effects by inhibiting multiple growth factors including TGF-β1, Wnt/β-catenin and insulin-like growth factor-1 (IGF-1) [8, 17, 27]. Administration of secreted klotho or genetic manipulation induces klotho overexpression to ameliorate renal fibrosis [17, 28].

The present study clearly showed that serum klotho level and klotho expression in kidney tissues were decreased in the mild form of tubulointerstitial fibrosis, and showed a consistently downward trend with progressive fibrosis. Serum klotho was also negatively correlated with the degree of segmental sclerosis. Furthermore, serum klotho level showed good discriminative ability to predict interstitial fibrosis and segmental sclerosis. Due to the high sensitivity of serum klotho for predicting renal fibrosis, we anticipate the absence of renal fibrosis at elevated serum klotho levels (cut-off value, 138.1 pg/mL). One study has reported that a down-regulation of klotho in renal tissue and peripheral blood mononuclear cell assessed by klotho promoter methylation is associated with tubulointerstitial fibrosis [29]. This suggests that klotho has an anti-fibrotic role in human renal interstitium, consistent with our results.

Our previous study reported that klotho deficiency was also associated with albuminuria, particularly in patients with diabetes [21]. While klotho expression is mainly observed in renal tubular cells, glomerulus is considered another site of klotho expression, and klotho deficiency has been associated with disruption of glomerular filtration [23]. Our study showed that UKCR was negatively correlated with diffuse foot process effacement of podocyte and not with IFTA or glomerulosclerosis. The results suggest that UKCR may reflect podocyte injury status. At a level of 96.7 pg/mgCr, UKCR also yielded good discriminative ability to predict diffuse foot process effacement.

We also focused on the role of RAS in klotho-renal fibrosis axis, especially intrarenal RAS activity, which was assessed based on urinary angiotensinogen [30]. Other studies reported that angiotensin inhibited klotho expression and klotho inhibited RAS activation [20, 31]. Therefore, it is plausible that soluble klotho and urinary angiotensinogen are negatively correlated. However in the present study, urinary angiotensinogen was not correlated with serum and urinary klotho, as well as renal expression of klotho. Urinary angiotensinogen was also not adequately correlated with the degree of renal fibrosis and other pathological findings in our study, although other studies suggested that urinary angiotensinogen represented a potential marker of deterioration of kidney function [32].

The level of serum klotho (14.0 [7.0–122.2] pg/mL) in the present study was significantly lower than in a previous study (580.2 [450.0–714.8], *P* < 0.001) [21]. On the other hand, the UKCR levels were 76.6 (12.1–197.2) pg/mL in the present study compared with 14.9 (39.2–286.7) pg/mL in the previous study (*P* = 0.803) [21]. In another study of serum klotho levels in CKD patients, the mean serum klotho level was 519 ± 183 pg/mL [33]. We attributed the discrepancy to active renal pathology of the participants in the present study, which inhibited the klotho expression. It was reported that fibrosis per se also inhibits kidney klotho expression via TNF superfamily inflammatory cytokines, TGF-β1, or angiotensin II [8, 27, 31, 34].

There are a few limitations in this study. First, the present study was cross-sectional in design. As renal biopsy and measurement of soluble klotho were conducted only at baseline, we could not determine the causality between klotho deficiency and changes in renal pathology. Monitoring the dynamic changes in biopsy findings and soluble klotho levels after appropriate treatment is essential to understand the mechanisms involved. Second, IHC staining for the detection of klotho protein was based only on a small number of patients. A certain trend between tissue klotho expression and pathologic findings was evident, but there was no statistical significance. Finally, the participants were limited to patients with glomerulonephritis and the findings may not be generalized to other renal conditions such as diabetic nephropathy, transplanted kidney, or other forms of CKD.

In conclusion, IFTA and segmental glomerulosclerosis in the biopsy specimens of human kidneys were associated with decreased serum klotho, and foot process effacement of podocyte was associated with decreased urinary klotho. Serum and urinary klotho showed favorable discriminative ability for predicting renal fibrosis and diffuse foot process effacement, respectively. The results highlight the potential of soluble klotho as a biomarker of renal fibrosis and podocyte injury in humans. The measurement of serum/urinary klotho ratio facilitates the sequential evaluation of renal disease progression in patients who are not available for renal biopsy.

## Supporting information

S1 Appendix Protocols for light microscopy, immunofluorescence, and electron microscopy. (DOCX)
