# Supplementary material for: Soluble klotho as a marker of renal fibrosis and podocyte injuries in human kidneys
Source: PLoS One. 2018 Mar 28;13(3):e0194617. doi: 10.1371/journal.pone.0194617 (PMC5874023; doi:10.1371/journal.pone.0194617)
Supplement: S1 Appendix — (DOCX) [file pone.0194617.s001.docx]

**S1 Appendix. Protocols for light microscopy, immunofluorescence, and electron microscopy**

For light microscopy, the specimens were fixed in Dubosq-Brazil solution, embedded in paraffin, and cut into 2-μm thick sections. The serial sections were stained with hematoxylin and eosin, periodic acid-Schiff, Masson’s trichrome, and Jones’ methenamine silver.

An immunofluorescence investigation was performed on the 2-μm thick sections that were obtained from snap-frozen tissues, which were incubated with immunoglobulins (IgG, IgA, and IgM), C1q, C3, light chains and fibrinogen antibodies.

For electron microscopy, kidney specimens were fixed in 2.5% glutaraldehyde, postfixed in 1% osmium tetroxide, and embedded in Epon mixture. These specimens were thin sectioned, treated with lead citrate/uranyl acetate, and examined using a JEM-100CX II transmission electron microscope (JEOL, Tokyo, Japan). Electron micrographs of one to three glomeruli per kidney were randomly taken at magnification from ×4,000 to ×40,000.
